# Selection of Antibiotics as Prophylaxis for Close Contacts of Patients with Meningococcal Disease in Areas with Ciprofloxacin Resistance — United States, 2024

**DOI:** 10.15585/mmwr.mm7305a2

**Published:** 2024-02-08

**Authors:** Isha Berry, Amy B. Rubis, Rebecca L. Howie, Shalabh Sharma, Daya Marasini, Henju Marjuki, Samuel Crowe, Lucy A. McNamara

**Affiliations:** ^1^Division of Bacterial Diseases, National Center for Immunization and Respiratory Diseases, CDC; ^2^Epidemic Intelligence Service, CDC.

**Summary**

**What is already known about this topic?**

Meningococcal disease cases caused by ciprofloxacin-resistant strains of *Neisseria meningitidis* have increased in the United States. Use of ciprofloxacin for antibiotic prophylaxis in areas with ciprofloxacin resistance might result in prophylaxis failure.

**What is added by this report?**

CDC provides implementation guidance for health departments for the preferential use of other recommended prophylaxis options (i.e., rifampin, ceftriaxone, or azithromycin) in place of ciprofloxacin when two or more ciprofloxacin-resistant meningococcal disease cases that account for ≥20% of all cases are reported in a local catchment area during a 12-month period.

**What are the implications for public health practice?**

Monitoring for prophylaxis failures and antimicrobial resistance among meningococcal isolates is essential to support the need for additional updates to recommendations.

## Abstract

Meningococcal disease, caused by the bacterium *Neisseria meningitidis*, is a rare but life-threatening illness that requires prompt antibiotic treatment for patients and antibiotic prophylaxis for their close contacts. Historically, *N. meningitidis* isolates in the United States have been largely susceptible to the antibiotics recommended for prophylaxis, including ciprofloxacin. Since 2019, however, the number of meningococcal disease cases caused by ciprofloxacin-resistant strains has increased. Antibiotic prophylaxis with ciprofloxacin in areas with ciprofloxacin resistance might result in prophylaxis failure. Health departments should preferentially consider using antibiotics other than ciprofloxacin as prophylaxis for close contacts when both of the following criteria have been met in a local catchment area during a rolling 12-month period: 1) the reporting of two or more invasive meningococcal disease cases caused by ciprofloxacin-resistant strains, and 2) ≥20% of all reported invasive meningococcal disease cases are caused by ciprofloxacin-resistant strains. Other than ciprofloxacin, alternative recommended antibiotic options include rifampin, ceftriaxone, or azithromycin. Ongoing monitoring for antibiotic resistance of meningococcal isolates through surveillance and health care providers’ reporting of prophylaxis failures will guide future updates to prophylaxis considerations and recommendations.

## Introduction

*Neisseria meningitidis* causes invasive meningococcal disease, a severe and life-threatening illness. Close contacts of patients with invasive meningococcal disease are at increased risk for acquiring the disease, and antibiotic prophylaxis is recommended for these persons. First-line options for prophylaxis are rifampin, ciprofloxacin, and ceftriaxone; azithromycin can also be used in areas with ciprofloxacin-resistant strains (*1*). Historically, antibiotic resistance in *N. meningitidis* has been uncommon in the United States (*2*). However, in 2020, CDC identified 11 ciprofloxacin- and penicillin-resistant *N. meningitidis* serogroup Y (NmY) isolates from cases occurring in 2019 and 2020 (*3*,*4*).

More recent data show that 29 cases caused by ciprofloxacin-resistant strains were reported during 2019–2021: 24 NmY (also resistant to penicillin), four NmB, and one nongroupable strain. No direct epidemiologic linkages among cases were identified. The median patient age was 24 years (range = 2 months–88 years) and 20 (69%) cases occurred among Hispanic or Latino persons; one case (3%) was fatal.

Although no instances of prophylaxis failure associated with ciprofloxacin resistance in the United States have been reported to date, use of ciprofloxacin as prophylaxis in areas with ciprofloxacin resistance might increase the likelihood of failure. Based on emerging evidence, CDC is providing updated guidance for health departments to aid in making decisions about when and where recommended antibiotic options other than ciprofloxacin should be preferentially considered for use as prophylaxis for close contacts of patients with invasive meningococcal disease.

## Methods

CDC considered four main criteria in developing the guidance for preferentially considering options other than ciprofloxacin for meningococcal disease prophylaxis. These include 1) a threshold for action (i.e., the number and percentage of cases caused by ciprofloxacin-resistant strains in a specified area and period, after which alternatives to ciprofloxacin should be preferentially considered), 2) the alternative antibiotics that should be used, 3) the duration of the guidance, and 4) the catchment area (i.e., the area in which cases are counted for determining the threshold and that will follow the changes in prophylaxis prescribing practices).

During October 2022–April 2023, these four criteria, as well as five contextual considerations (acceptability to public health partners, feasibility in implementation, effect on health equity, potential indirect outcomes, and anticipated opposition), were evaluated using an iterative process. CDC began by soliciting feedback on the criteria and contextual considerations from governmental and nongovernmental subject matter experts, including experts from within the agency, jurisdictional health departments, and academic institutions, to gain information on the need for updated guidance and to discuss the practical considerations that could affect guidance implementation. CDC experts developed draft implementation guidance, after which additional feedback was solicited from state and local public health professionals who would potentially implement this guidance. This feedback was considered by CDC when formulating the final guidance.

## Rationale and Evidence

### Invasive Meningococcal Disease Cases and Resistance Patterns

**FIGURE 1:**
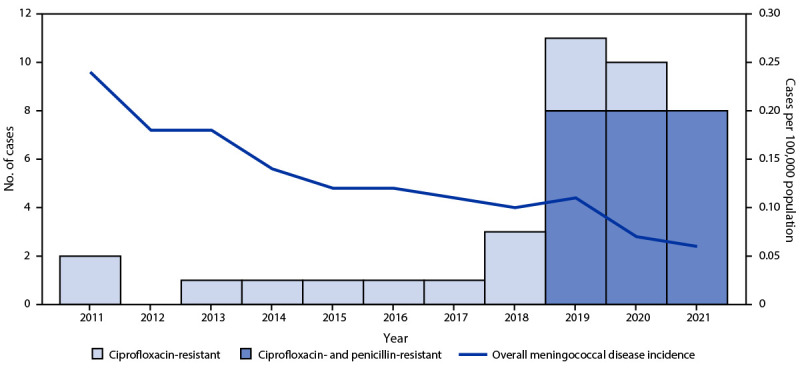
Meningococcal disease incidence and number of invasive meningococcal disease cases caused by ciprofloxacin-resistant or ciprofloxacin- and penicillin-resistant strains of *Neisseria meningitidis* — United States, 2011–2021

**FIGURE 2:**
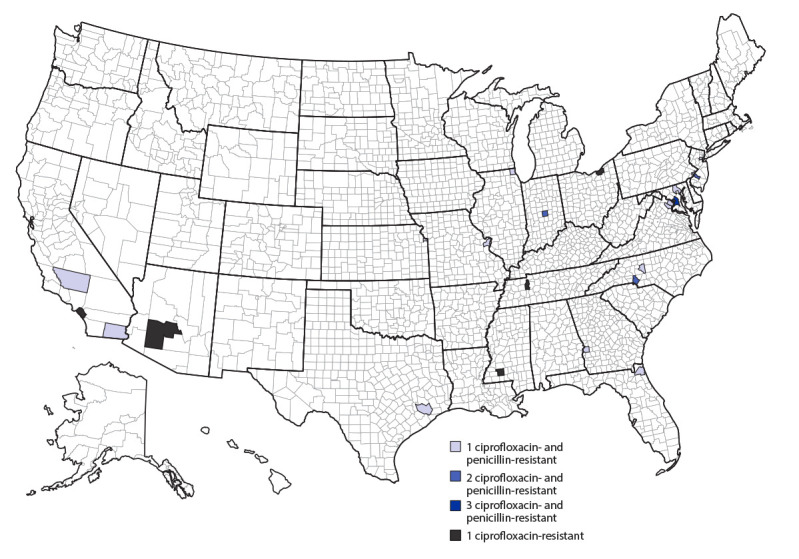
Number of invasive meningococcal disease cases caused by ciprofloxacin-resistant or ciprofloxacin- and penicillin-resistant *Neisseria meningitidis* strains, by county — United States, 2019–2021

An annual average of 1.25 cases of invasive meningococcal disease caused by ciprofloxacin-resistant strains were reported in the United States during 2011–2018; however, the number of such cases has increased sharply since 2019. An annual average of 9.7 cases of invasive meningococcal disease caused by ciprofloxacin-resistant strains were reported in 2019, 2020, and 2021, despite an overall 75% decline in disease incidence from 0.24 cases per 100,000 population (2011) to 0.06 (2021) (Figure 1). Recent cases were predominantly caused by ciprofloxacin- and penicillin-resistant NmY strains and were distributed across the United States, but clusters were identified in some geographic areas (Figure 2).

### Considerations in Determining Resistance Thresholds

Resistance thresholds for recommending changing antibiotics are inconsistent across pathogens and contexts (*5*). CDC experts agreed that, because of the severity of invasive meningococcal disease and high mortality risk in potential instances of prophylaxis failure, the threshold should be low. In determining the threshold for action, both a specific number of resistant cases (e.g., one or two) and a percentage (e.g., 20%) of all cases were needed to allow sufficient flexibility for jurisdictions with high invasive meningococcal disease incidence to act while ensuring areas with low incidence were not changing recommendations based on a single, potentially sporadic, resistant case.

Existing guidance states that rifampin (4 oral doses in 48 hours), ciprofloxacin (single oral dose), or ceftriaxone (single injection) are first-line antibiotics for meningococcal prophylaxis; a single oral dose of azithromycin has also been used in areas with ciprofloxacin-resistant strains (*1*). A published systematic review and meta-analysis determining effectiveness, adverse events, and development of drug resistance for different meningococcal prophylaxis regimens was used as supporting evidence for determining when to favor the use of recommended prophylaxis options other than ciprofloxacin (*6*). Six studies presented data on rifampin compared with placebo and found that rifampin was effective at eradicating *N. meningitidis* 1 week after prophylaxis (meta-analysis pooled risk ratio [RR] = 0.17; 95% CI = 0.13–0.24) (*6*). No trials evaluated ceftriaxone or azithromycin against placebo, but two studies comparing rifampin with ceftriaxone found no statistically significant difference in eradication (RR = 3.71; 95% CI = 0.73–18.86) (*6*), and one study comparing azithromycin to rifampin reported no statistically significant difference in eradication (RR = 0.30; 95% CI = 0.30–5.54) (*6*,*7*). Across nine studies examining side effects and adverse events for at least one of the alternative antibiotics, reported adverse events were mild and included nausea, diarrhea, abdominal pain, headaches, dizziness, and skin rashes. Compared with rifampin, one study found a higher adverse event rate with ceftriaxone (RR = 1.39; 95% CI = 1.10–1.75); however, this difference was primarily driven by reports of pain at the injection site. Six studies reported on the antibiotic susceptibility of persistent isolates to at least one of the alternative antibiotics; development of resistance following prophylaxis was detected only for rifampin (*6*). Resistance to rifampin has also been reported in mass chemoprophylaxis settings, but because there is a fitness cost to the mutations associated with resistance, resistant strains have not become widespread (*8*); occasional rifampin prophylaxis failures have also been reported (*9*). CDC experts reviewed the literature since 2013 for updated data on the effectiveness of alternative prophylaxis regimens; no new data were identified.

The CDC expert group also considered adherence, acceptability, contraindications, and dosing regimens for the alternative antibiotics and noted that despite limited evidence of effectiveness, azithromycin would likely be the logistically simplest replacement for ciprofloxacin among the existing recommended prophylaxis options. In determining the duration of guidance, feasibility and communication challenges were considered, recognizing that frequent changes in recommended prophylaxis antibiotics within a local area might cause confusion among providers and public health staff members and might lead to lack of adherence. Flexibility in guidance criteria to allow for unique jurisdictional and cross-jurisdictional considerations during implementation, particularly when defining a catchment area, was emphasized in feedback discussions.

## Presentation of Guidance

### Implementation Guidance for Health Departments

Based on the currently recommended prophylaxis options (*1*), the 2013 systematic review (*6*), and expert feedback using the stated criteria and contextual considerations, the implementation guidance for health departments includes the circumstances under which ciprofloxacin prophylaxis should be discontinued and alternative antibiotic prophylaxis options should be preferentially considered, alternative prophylaxis regimens, and the extent and duration of implementation of the updated guidance (Box).

BOX **Implementation guidance for health departments for preferentially considering antibiotics other than ciprofloxacin for invasive meningococcal disease prophylaxis**

Discontinue use of ciprofloxacin as prophylaxis for close contacts when both of the following threshold criteria have been met in the catchment area* during a rolling 12-month period:

- Two or more invasive meningococcal disease cases caused by ciprofloxacin-resistant strains have been reported, and
- Cases caused by ciprofloxacin-resistant strains account for ≥20% of all reported invasive meningococcal disease cases.

Prescribe rifampin, ceftriaxone, or azithromycin instead of ciprofloxacin as prophylaxis when the threshold criteria have been reached.^†^

Implement updated prophylaxis guidance in all counties within the catchment area.

Maintain updated prophylaxis guidance until a full 24 months have passed without any invasive meningococcal disease cases caused by ciprofloxacin-resistant strains having been reported in the catchment area.

* The catchment area should be a single contiguous area that contains all counties reporting ciprofloxacin-resistant cases. Jurisdictions should include surrounding counties, if warranted, based on population mixing patterns.

^†^ https://www.cdc.gov/vaccines/pubs/surv-manual/chpt08-mening.html

Health departments have flexibility in guidance implementation. Updated prophylaxis guidance can be implemented at a lower threshold or extended across a broader area, such as across a metropolitan statistical area or health department catchment area. Other health department considerations in determining guidance implementation include local epidemiology; feasibility (e.g., logistical simplicity of having a particular geographic area follow uniform guidance); epidemiologic linkages among patients; travel history, including college and other students’ travel to or from school*; and patterns in population movement, including movement across jurisdictional borders.

### Benefits and Harms

The primary anticipated public health benefit of this guidance is a reduced likelihood of ciprofloxacin prophylaxis failure. However, potential prophylaxis failures with alternative antibiotics might occur, and the potential for reduced adherence or slower administration of less convenient alternative prophylaxis options remains.

## Discussion

CDC’s implementation guidance for choosing antibiotics for invasive meningococcal disease prophylaxis is based on observed increases in the number of cases of invasive meningococcal disease caused by ciprofloxacin-resistant strains since 2019 and concerns about potential prophylaxis failures in areas with ciprofloxacin resistance. These data, combined with evidence that alternative recommended prophylaxis options are effective and are associated with minimal adverse events, support preferentially considering the use of antibiotics other than ciprofloxacin in areas reaching a minimum threshold for action.

Antimicrobial susceptibility testing for *N. meningitidis* is typically conducted at CDC rather than locally and is not routinely conducted in support of patient care. Therefore, results to guide prophylaxis options for close contacts of individual cases are often not available. However, if antimicrobial susceptibility testing results demonstrating resistance in an index patient are promptly available by local testing, adjustments in prophylaxis can also be made, regardless of whether a local area has reached the recommended threshold.

Effective guidance implementation will depend on rapid communication of antimicrobial susceptibility testing results between CDC and jurisdictions to guide local threshold calculations, strong cross-jurisdictional communication regarding catchment area borders, availability of alternative antibiotics, and monitoring for potential prophylaxis failures. A need remains to generate more data on azithromycin’s effectiveness because it is likely the most convenient and readily available alternative antibiotic for meningococcal prophylaxis.

CDC staff members are available to provide technical assistance if questions about guidance implementation arise. To support monitoring and evaluation of guidance implementation, health departments are requested to notify CDC about any changes made to prophylaxis guidance at meningnet@cdc.gov. CDC will continue to monitor for prophylaxis failures and antimicrobial resistance among meningococcal isolates to determine whether adjustments are needed and will update the guidance as new data become available.

## Footnotes

*
https://learn.cste.org/images/dH42Qhmof6nEbdvwIIL6F4zvNjU1NzA0MjAxMTUy/Course_Content/Case_based_Surveillance_for_Syphilis/CSTE_Revised_Guidelines_for_Determining_Residency_for_Disease_Reporting_Purposes.pdf
